# Androgen receptor is expressed in mouse cardiomyocytes at prenatal and early postnatal developmental stages

**DOI:** 10.1186/s12899-017-0033-8

**Published:** 2017-08-14

**Authors:** Enrique Pedernera, María José Gómora, Iván Meneses, Marlon De Ita, Carmen Méndez

**Affiliations:** Facultad de Medicina, Edificio E, Universidad Nacional Autónoma de México, Av. Universidad #3000, Coyoacán, 04510 Cd. de México, CP Mexico

**Keywords:** Androgen receptor, Mouse embryo, Cardiac myocytes, Atrial natriuretic factor, Heart development

## Abstract

**Background:**

Previous studies show that androgens are involved in hypertrophy and excitability of cardiomyocytes and that their effects are mediated through their receptor. The aim of this study was to evaluate the presence of androgen receptor (AR) in mouse heart during prenatal and early postnatal stages.

**Results:**

The expression of AR and related genes, alpha myosin heavy chain -Myh6-, beta myosin heavy chain -Myh7- and atrial natriuretic factor –Nppa- was simultaneously evaluated by semiquantitative RT-PCR. AR was also detected by immunohistochemistry. Androgen receptor mRNA was detected in hearts from 10.5 days post coitum to 16 postnatal days. A higher expression of AR mRNA in atria compared to ventricles was observed in neonatal mouse. A positive correlation between mRNA levels of AR and Nppa was observed in mouse heart at early postnatal development. Androgen receptor expression is similar in males and females during cardiac development. Finally, androgen receptor protein was observed by immunohistochemistry in myocardial cells of atria and ventricles from 12.5 days onwards and restricted after 16.5 days post-coitum to nuclei of cardiomyocytes.

**Conclusion:**

Present results provide evidence that androgen receptor is expressed from prenatal stages in mouse heart, supporting the proposition that androgens could be involved in mammalian heart development.

## Background

The involvement of androgens in gender-related cardiovascular diseases [1] explains the interest for the study of the role of sexual steroids on cardiac myocytes. There are sex-related differences in mRNA expression of alpha- and beta-myosin heavy chains (MHC) and other functional proteins in rat myocardium [2]. The MHC composition changes in ventricular myocytes of castrated rats and it is restored by testosterone treatment [3]. Moreover, androgens influence the expression of genes regulating intracellular calcium and contractile performance of ventricular myocytes in postnatal rats [4]. A sex-related difference in the cardiac response to atrial natriuretic peptide has been described in spontaneously hypertensive rats. On the other hand, atrial natriuretic peptide is differentially expressed between atria and ventricles in the human heart and has been related to cardiac hypertrophy and remodeling [5–7].

The presence of androgen receptor in embryonic heart would be important to indicate a role of androgens in prenatal cardiac development. The aim of this work was to determine the expression of androgen receptor simultaneously with the expression of alpha and beta myosin heavy chain genes (Myh6 and Myh7) and atrial natriuretic peptide gene (Nppa) at prenatal and early postnatal stages of mouse heart development. The presence of mRNA and the protein of the androgen receptor was observed in the nuclei of cardiac myocytes from embryonic stages and a positive correlation between AR and Nppa mRNA’s was registered at 2 and 9 postnatal days.

## Methods

### Animals

CD1 mice were caged with food and water ad libitum under a 12-h light/12-h dark cycle in a room with a constant temperature of 23 ± 2 °C. Female mice were mated with males overnight, when vaginal plug was found, it was determined as 0.5 days post coitum (dpc). Pregnant females from 8.5, 10.5, 12.5, 14.5, 16.5 and 18.5 dpc were euthanized by cervical dislocation to obtain the embryos. The developmental stage was corroborated according to characteristics described at *emouseatlas.org*. Mouse pups from 2, 9 and 16 postnatal days (pnd) were euthanized by decapitation. The sex of embryos and pups was determined after 12.5 dpc by gonad examination. All the procedures were approved by the Ethics and Research Committee of the Facultad de Medicina, Universidad Nacional Autónoma de México (UNAM) and according to the National Institutes Health guidelines.

### Total RNA purification and RT-PCR analysis

Atria and ventricles were obtained at pre-and postnatal stages and directly stored in RNA later solution (Qiagen, Valencia, CA). Samples of males and females were pooled separately to obtain enough tissue; at least three hearts were included in each pool. Total RNA was isolated using Trizol (Life Technologies, Gaithersburg, MD) according to manufacturer’s instructions. Total RNA was measured using NanoDrop (Thermo Scientific, Barrington, IL). Total RNA (1.0 μg) was reversely transcribed to cDNA by Transcriptor Reverse Transcriptase (Roche) in 20 μL reaction mixture. For semi-quantitative PCR analysis 1 μL of cDNA as template was amplified with Platinum Taq DNA polymerase (Invitrogen, Carlsbad, CA) in 20 μL PCR reactions. The number of cycles was selected to be in the linear portion of the exponential curve. The sequences of primers used were: for mouse beta-actin (NM_007393) sense primer sequence: 5′-gtatgcctctggtcgtacca-3′ and antisense primer sequence: 5′-ttgctgacaggatgcagaag-3′; mouse glyceraldehyde-3-phosphate dehydrogenase (NM_008084) sense primer sequence: 5′-atggtgaaggtcggtgtgaa −3′ and antisense primer sequence: 5′-gattgtcagcaatgcatcctgc-3′; mouse androgen receptor (NM_013476) sense primer sequence: 5′-gagtgactactctgcctccgaag-3′ and antisense primer sequence: 5′-gttatgaagcagggatgactctggg-3′; mouse myosin, heavy polypeptide 6, cardiac muscle, alpha (NM_001164171)sense primer sequence: 5′-atctctgacaacgcctatc-3′ and antisense primer sequence: 5′-gataggcgttgtcagagat-3′; mouse myosin, heavy polypeptide 7, cardiac muscle, beta (NM_080728) sense primer sequence: 5′-tgtgctgtacaacctcaagg-3′ and antisense primer sequence: 5′-ccttgaggttgtacagcaca-3′; mouse natriuretic peptide type A (NM_008725) sense primer sequence: 5′-aataaacttcagcaccaaggac-3′ and antisense primer sequence: 5′-gtccttggtgctgaagtttatt-3′. The PCR products were size-fractionated by 1% agarose gel electrophoresis and visualized with ethidium bromide using an EpiChemi II Darkroom (UVP Inc., Upland, CA). PCR bands were subjected to densitometry analysis with Quantity One software (Bio-Rad). Measurements were done by triplicates and the average was registered as the value of the sample. Data were normalized to beta-actin values, which were obtained simultaneously in each sample, before statistical analysis. The sequence determination was carried out using the Automated DNA sequencer model 373 (PE- Applied Biosystems Foster CA).

### Immunohistochemistry

Complete embryos before 14.5 dpc and hearts from older time points were fixed by immersion overnight in saline phosphate buffer containing 4% paraformaldehyde and paraffin embedded. Tissue sections 3 μm thick, were deparaffinized and rehydrated. Antigen retrieval was carried out in a pressure chamber for 5 min in Diva decloaker citrate buffer (Biocare, Pike Lane Concord, CA). Non-specific binding sites were blocked with 10% goat serum for 1 h at room temperature. Tissue slices were incubated overnight at 4 °C with anti-AR polyclonal antibody diluted 1:50 (Santa Cruz Biotechnology, Santa Cruz, CA). AR antibody evaluated by Western blot technique binds to a 110 kDa protein, the expected size of androgen receptor. Slides were further incubated with Mach2 rabbit HRP polymer (Biocare) for 1 h at room temperature. Signal detection was achieved with diaminobencidin chromogen kit (Biocare). Color development was stopped by PBS rinsing and counterstained with Gill’s hematoxylin. Samples without first AR antibody were used as negative controls. Histological sections of testis were used as positive controls.

### Statistical analysis

Data were analyzed by ANOVA and *post-hoc* Tukey test, Student’s *t* test and Pearson correlation coefficient as indicated in figures. Results were considered significant when *P* values were less than 0.05.

## Results

**Fig. 1:**
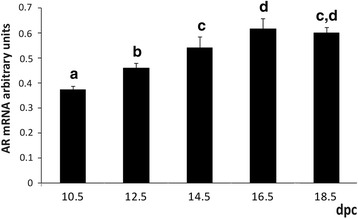
Androgen receptor mRNA levels in prenatal heart development. Bars represent AR mRNA expressed in arbitrary density units, mean ± SD (*n* = 4–6); a different superscript indicates statistical significance after ANOVA and *post-hoc* Tukey test

Androgen receptor mRNA was evaluated by semi quantitative PCR in the heart of mouse embryo; the presence of AR mRNA was observed from 10.5 dpc until birth. The AR expression increases gradually with the highest levels registered at 16.5–18.5 dpc (Fig. 1).

**Fig. 2:**
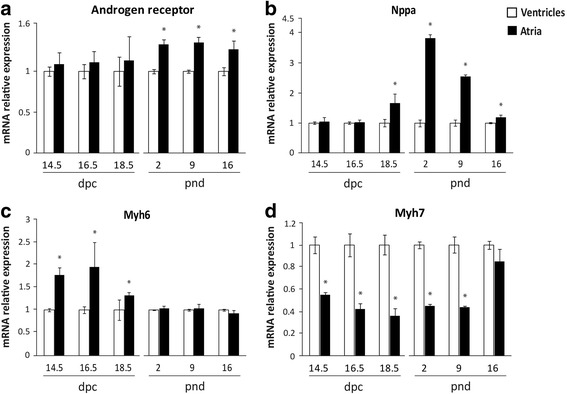
Expression of **​a** androgen receptor, **​b** Nppa, **c** Myh6 and **​d** ​Myh7 in atria and ventricles of prenatal and postnatal mouse heart. Measurements were obtained in arbitrary density units relative to beta-actin. Graphs show relative expressions at pre-and postnatal developmental stages, normalized with the mean value obtained in ventricles at each developmental stage. Data are expressed as an average of the triplicates. Bars represent mean ± SD (*n* = 4–6), * *p* < 0.05

Temporal changes in the expression of AR mRNA were simultaneously evaluated with Myh6, Myh7 and Nppa in atria and ventricles of prenatal and postnatal hearts. The presence of AR mRNA was similar in atria and ventricles at 14.5, 16.5, 18.5 dpc in prenatal heart development. In postnatal hearts at 2, 9 and 16 pnd, a higher expression of AR mRNA in atria was observed compared with that of the age-matched ventricles (Fig. 2a). Similarly, Nppa expression was significantly higher in atria compared to that of ventricles from 18.5 dpc to 16 pnd (Fig. 2b). The expression of Myh6 was significantly higher in atria than in ventricles at 14.5–18.5 dpc prenatal stages (Fig. 2c). On the other hand, Myh7 is highly expressed in ventricles from prenatal and postnatal developmental stages. A similar expression of Myh7 between chambers was registered at 16 pnd (Fig. 2d).

**Fig. 3:**
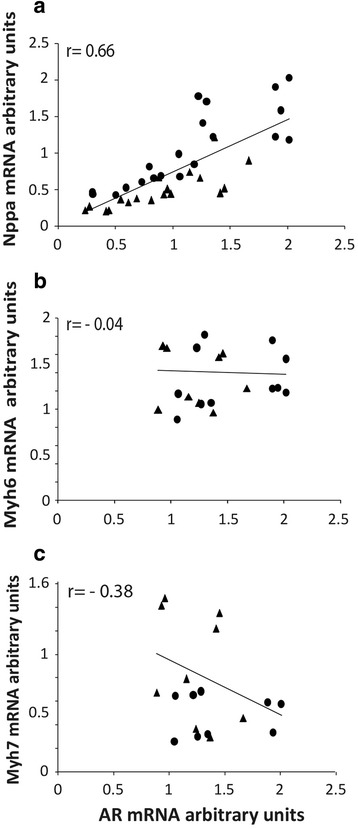
Comparison of mARN levels of AR, with Nppa, Myh6 and Myh7. Correlations are represented in atria (*circles*) and ventricles (*triangles*) of mouse heart at 2 and 9 pnd. Values for each gene are expressed in arbitrary density units. Lineal regression and correlation index (Pearson) are indicated (**a**, **b**, **c**). Correlation index separated by age and heart chambers are indicated in results

Androgen receptor mRNA displayed a significant correlation with the expression of Nppa at 2 and 9 dpn heart development (Fig. 3a) but not with Myh6 or Myh7 (Fig. 3b, c). Correlation of AR with Nppa was also observed at each age, separately; at 2 pnd “r” value was 0.78 (*n* = 24); at 9 pnd *r* = 0.75 (*n* = 20). Moreover, AR correlates with Nppa in atria (*r* = 0.67, *n* = 22) and ventricles (*r* = 0.71, *n* = 22) indicating that correlation is observed in both chambers despite the highest level of Nppa observed in atria. There was no correlation of AR with Nppa, Myh6 or Myh7 in prenatal stages.

**Fig. 4:**
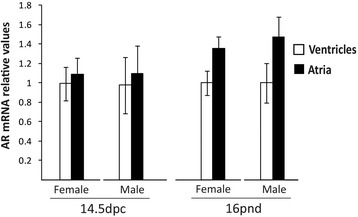
Androgen receptor mRNA relative expression at 14.5 dpc and 16 pnd in male and female mice. Relative expression values are normalized with the mean value obtained in ventricles at each developmental stage. Bars represent mean ± SD (*n* = 4–6)

Androgen receptor mRNA was similarly expressed in male and female hearts, independently of the age and the chamber studied. Androgen receptor mRNA values determined in atria and ventricles at 14.5 dpc, and 16 pnd separated by gender is shown in Fig. 4.

**Fig. 5:**
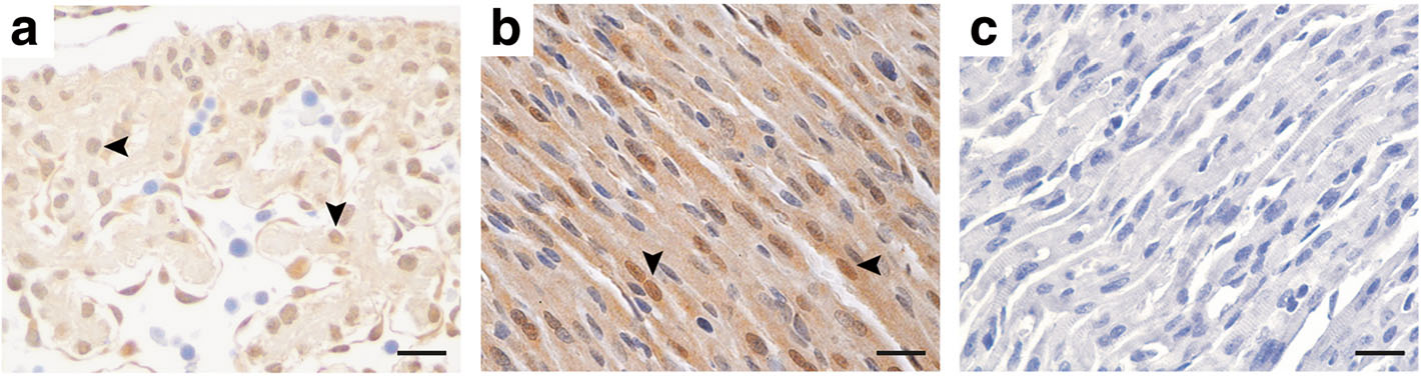
Androgen receptor detection by immunohistochemistry in prenatal and postnatal heart development. Representative images of **a** 12.5 dpc, and **b** 9 pnd stage. The AR immunolabeling is present in cardiac myocyte nuclei (*arrow heads*) but surrounding cells are negative. **c** Negative control. The scale bar represents 5 μm

Immunoreactivity for androgen receptor was evaluated in heart samples obtained at 12.5, 14.5, 16.5, 18.5 dpc, and 2, 9 and 16 pnd. The presence of the androgen receptor was detected in the cell nuclei of myocytes and endocardial cells at 12.5 dpc (Fig. 5a). At more advanced stages, 16.5 dpc onwards not all myocardial cells were positive for AR, images of positive and negative nuclei began to be identified. In postnatal hearts, we can distinguish cardiac myocytes from fibroblast by morphology; the presence of AR was limited to the nuclei of cardiac myocytes and fibroblast nuclei were negative (Fig. 5b). Fibroblasts localized at cardiac valves and fibrous skeleton of heart were AR negative (not shown). The expression of AR was similar in samples obtained at 2, 9 and 16 pnd. No differences were detected by immunohistochemistry between atria and ventricles.

## Discussion

Androgen receptor expression is herein described through prenatal and early postnatal heart development. We demonstrated that AR mRNA is present during the major stages of heart morphogenesis: looping heart (10.5 dpc) and chamber formation (12.5, 14.5 and 16.5 dpc); AR is also detected during heart functional maturation (18.5 dpc, 2, 9 and 16 pnd). Expression of AR was similar in males and females in the evaluated developmental stages. The presence of AR is higher in atria than in ventricles at 2, 9 and 16 pnd and mRNA levels for AR correlates with Nppa levels at 2 and 9 pnd of postnatal heart development.

The role of androgens in heart development has been previously analyzed; testosterone and dihydrotestosterone, acting through androgen receptor, induce the differentiation of stem cells into beating cardiac myocytes [8, 9]. Moreover, testosterone recruits AR to the regulatory regions of MEF2C and HCN4 genes in mouse embryonic stem cells [9]. Additionally, treatment with anti-androgenic compounds supports the requirement of the AR genomic pathway [9]. However, becoming a specialized cardiac myocyte is a complex ordered process that continues even after birth [10]. A previous study has demonstrated the presence of AR in the heart of newborn mammals and humans [11]. Present results demonstrate the presence of AR at early developmental stages, from 10.5 dpc after cardiac cell lineage establishment [12], suggesting the participation of androgen receptor in terminal differentiation of cardiac myocytes.

Moreover, present results show that AR displays a higher expression in atria than in ventricles at 2, 9 and 16 days after birth. In neonatal mouse, cardiac myocytes perform terminal differentiation and display ion channel expression profiles distinct from that of the adult mouse [13]. AR knock-out mouse shows altered atrium electrophysiology due to calcium protein dysregulation [14]^.^ Similarly, in adult rat atria, calcium-handling proteins from sarco-endoplasmic reticulum were altered after orchiectomy and prevented by testosterone replacement [15]. These results suggest that androgens can regulate ion channel expression during atrium postnatal development, but this proposal merits further studies.

During heart development, there is a dynamic expression of several genes, included the alpha myosin heavy chain, Myh6, beta myosin heavy chain, Myh7, and atrial natriuretic factor A, Nppa [16, 17]. This study confirms that Myh6 transcript is preferentially expressed in atria before birth; afterwards, the mRNA levels in atria and ventricle became similar. Expression of Myh7 predominates in ventricle at the evaluated developmental stages. Moreover, the ratio of Myh6/Myh7 is similar to that of previous reports [16]. Additionally, we have observed that mRNA of Nppa predominates in atria at 2, 9 and 16 pnd. AR shows a significant correlation with mRNA Nppa expression in atria and ventricles at perinatal stages; these results are not observed for Myh6 or Myh7. These findings could be explained because both AR and Nppa have a hypothetical upstream common regulator gene. Alternatively, it suggests a direct relationship between AR and Nppa expression. It has been reported that dihydrotestosterone increases ANP production in rat neonatal cardiac myocytes and AR antagonist treatment with cyproterone abolishes the effect of dihydrotestosterone on ANP secretion [11]. Further studies will be required to corroborate an androgenic regulation of Nppa expression in cardiac development.

No variations in the expression of AR between males and females are herein detected, either at prenatal or postnatal stages. Similarly, it has been reported that AR did not differ between male and female in the cytosolic and nuclear fractions of adult mouse ventricles and atria [18]. There are differences between genders in the adult normal heart physiology, and androgens induce changes in the heart of adult male mammals, including cardiac mass and mitochondrial function [19–22].

The immunoreactivity for AR protein is displayed in nuclei of atrial and ventricular myocardium from 12.5 dpc to 16 pnd. The presence in the nuclei of the heart cells suggests that AR is an active transcription factor. Myocardial and endocardial cells are positive for AR at 12.5 dpc. Meanwhile, at postnatal stages, it is clearly identified that cardiac myocytes are positive while cardiac fibroblasts are negative. Previous studies have described the presence of androgen receptors in nuclear subcellular fraction of mouse cardiac myocytes [18]. Cardiac fibroblasts appear around embryonic day 12.5 and increase in number steadily through postnatal day one [23, 24]. Herein, a negative immunoreactivity for AR seems to be displayed in fibroblasts of endomysium.

## Conclusion

The androgen receptor is expressed during the morphogenesis and maturation of mouse heart, primarily restricted to cardiac myocytes. A high expression of AR in atrial tissue is observed at early postnatal heart development, together with a positive correlation between AR and Nppa expression in atria and ventricles. Present data support that androgen receptor action would be relevant in mammalian heart development.

## Abbreviations

ANP: atrial natriuretic peptide
AR: androgen receptor
HCN4: hyperpolarization activated cyclic nucleotide-gated
MEF2C: myocyte enhancer factor
MHC: myosin heavy chains
Myh6: alpha myosin heavy chain gene
Myh7: beta myosin heavy chain gene
Nppa: atrial natriuretic peptide gene
